# PHD3 Acts as Tumor Suppressor in Mouse Osteosarcoma and Influences Tumor Vascularization via PDGF-C Signaling

**DOI:** 10.3390/cancers10120496

**Published:** 2018-12-06

**Authors:** Antje Egners, Maryam Rezaei, Aleksandar Kuzmanov, David M. Poitz, Doreen Streichert, Thomas Müller-Reichert, Ben Wielockx, Georg Breier

**Affiliations:** 1 Department of General, Visceral and Transplantation Surgery, RWTH University Hospital, 52074 Aachen, Germany; aegners@ukaachen.de; 2 Department of Pathology, TU Dresden, 01307 Dresden, Germany; 3 Department of Biochemistry, University of Münster, 48149 Münster, Germany; mrezaei@uni-muenster.de; 4 Department of Dermatology, University Hospital Zurich, CH-8952 Schlieren, Switzerland; al.kuzmanov@yahoo.com; 5 Institute for Clinical Chemistry and Laboratory Medicine, TU Dresden, 01307 Dresden, Germany; david.poitz@tu-dresden.de; 6 Core Facility Cellular Imaging, Experimental Center, Faculty of Medicine, TU Dresden, 01307 Dresden, Germany; doreen.streichert@tu-dresden.de (D.S.); mueller-reichert@tu-dresden.de (T.M.-R.); 7 Institute for Clinical Chemistry and Laboratory Medicine, TU Dresden, 01307 Dresden, Germany; ben.wielockx@tu-dresden.de; 8 Division of Medical Biology, Department of Psychiatry and Psychotherapy, TU Dresden, 01307 Dresden, Germany

**Keywords:** prolyl hydroxylase domain protein, PDGF-C, tumor angiogenesis, tumor progression, PDGFR-α

## Abstract

Cancer cell proliferation and insufficient blood supply can lead to the development of hypoxic areas in the tumor tissue. The adaptation to the hypoxic environment is mediated by a transcriptional complex called hypoxia-inducible factor (HIF). HIF protein levels are tightly controlled by oxygen-dependent prolyl hydroxylase domain proteins (PHDs). However, the precise roles of these enzymes in tumor progression and their downstream signaling pathways are not fully characterized. Here, we study PHD3 function in murine experimental osteosarcoma. Unexpectedly, PHD3 silencing in LM8 cells affects neither HIF-1α protein levels, nor the expression of various HIF-1 target genes. Subcutaneous injection of PHD3-silenced tumor cells accelerated tumor progression and was accompanied by dramatic phenotypic changes in the tumor vasculature. Blood vessels in advanced PHD3-silenced tumors were enlarged whereas their density was greatly reduced. Examination of the molecular pathways underlying these alterations revealed that platelet-derived growth factor (PDGF)-C signaling is activated in the vasculature of PHD3-deficient tumors. Silencing of PDGF-C depleted tumor growth, increased vessel density and reduced vessel size. Our data show that PHD3 controls tumor growth and vessel architecture in LM8 osteosarcoma by regulating the PDGF-C pathway, and support the hypothesis that different members of the PHD family exert unique functions in tumors.

## 1. Introduction

The vasculature of tumors is often unable to keep pace with the rapidly proliferating tumor cells and displays structural and functional abnormalities, leading to an inadequate supply of oxygen and nutrients to the tissue [1]. In consequence, many human cancers develop areas of low oxygen tension (hypoxia). A multitude of adaptive responses are being induced by hypoxia, including alterations in tumor cell metabolism, survival and invasion, as well as the formation of new blood vessels (angiogenesis). At the molecular level, the hypoxia response is mediated primarily by hypoxia-inducible factor-1 (HIF-1), a heterodimeric transcription factor composed of an oxygen-dependent α-subunit and a constitutive β-subunit. Elevated HIF-1α levels were observed in different human cancer types and correlate with increased tumor aggressiveness, invasiveness [2,3] and often with negative overall survival rates and poor prognosis [4,5,6]. HIF-1 targets control processes that are crucial during tumor progression including genes that stimulate angiogenesis, cell survival, mobility, and glucose metabolism [7,8].

More than a decade ago, the intricate regulatory pathways that govern HIF abundance and activity were unraveled [9]. The HIF-α subunit is subject to post-translational modification on conserved proline or asparagine residues. In the presence of oxygen, prolyl-hydroxylase domain proteins (PHDs) catalyze the hydroxylation of HIF-1α or HIF-2α [10,11,12], causing their proteasomal degradation [13,14]. Factor inhibiting HIF-1 (FIH) diminishes the transcriptional activity of HIF-1 by suppressing its binding to transcriptional co-factors [15]. However, HIF-independent functions exerted by PHD proteins were also reported. Mitochondrial respiration is being regulated by PHD1 in ERα-positive breast cancer in a HIF-1-independent manner [16]. PHD2 regulates the secretion of interleukin-8 and angiogenin without the involvement of HIF-1 in HCT116 colon carcinoma cells [17] and it directly interacts with EGFR, influencing subsequent signaling events in breast cancer [18]. PHD3 overexpression in pancreatic cancer induces apoptosis HIF-independently [19]. Furthermore, PHD3 was reported to preferentially hydroxylate HIF-2α and to interact with several additional partners [20,21,22,23,24]. Modulation of fatty acid oxidation by PHD3 in the absence of HIF-1 activity was reported by German et al. [25].

Analyses of human malignancies have shown that the PHDs can be either up- or down-regulated in cancerous compared to healthy tissues [17,19,25,26,27,28,29]. The abundance of PHDs correlates with their overall catalytic activity [20] and thus, might influence tumor progression. Recent experimental evidence supports this hypothesis. Chan et al. reported that the loss of PHD2 in tumor cells increases the growth of human pancreatic adenocarcinoma and colon carcinoma xenografts in mice via enhanced angiogenesis [17]. Yet, opposite effects were observed in other tumor models. As demonstrated by us, PHD2 deficiency reduces the growth of murine LM8 osteosarcoma and Lewis lung carcinoma, by converting the tumor-promoting activity of transforming growth factor (TGF)-β into a growth-inhibitory activity [30]. While the role of PHD2, the main regulator of HIF stability [31], is well studied, less is known about the specific functions of the other PHD family members in tumors. A study by Miikkulainen et al. demonstrated crucial involvement of PHD3 in glucose metabolism, translation as well as mRNA processing in clear cell renal cell carcinoma [29]. PHD3 expression induced apoptosis in non-small cell lung cancer [32], but protected glioblastoma cells from hypoxia-induced cell death [33]. Tennant and Gottlieb demonstrate a direct link between PHD3 and cell viability in melanoma, squamous cell as well as colon carcinoma [34]. Overexpression of PHD3 in pancreatic cancer cell lines inhibited experimental tumor growth and angiogenesis [19], suggesting that PHD3 acts as tumor suppressor. Consistently, knockdown of PHD3 in colorectal tumor xenografts enhanced their growth in immunodeficient mice [27]. These authors showed that PHD3 inhibits IKKb/NF-kB signaling, independent of its hydroxylase activity. 

Here we have studied the role of PHD3 in cancer biology by an RNA-interference approach in a murine syngeneic tumor model. We report that silencing PHD3 in LM8 osteosarcoma cells with small hairpin RNA (shRNA) enhances tumor growth, and induces profound changes of the tumor vessel density, morphology and perivascular cell coverage. These alterations were caused by up-regulation of platelet-derived growth factor (PDGF)-C. Silencing PDGF-C expression in LM8-shPHD3 cells decreased tumor growth and reversed the vascular phenotype. Activated PDGF receptor-α (pPDGFR-α, Tyr754) was localized primarily on vascular cells, suggesting that the PDGF-C-induced effects are, at least in part, mediated by indirect mechanisms involving the activation of endothelial cells. Our data reveal a previously unknown link between PHD3 and PDGF-C in experimental osteosarcoma and provide novel information about the function of this pathway in regulating vascularization and progression of tumors.

## 2. Results

### 2.1. PHD3 Silencing Does not Affect HIF-1α Protein Levels or HIF-Target Gene Expression

**Figure 1:**
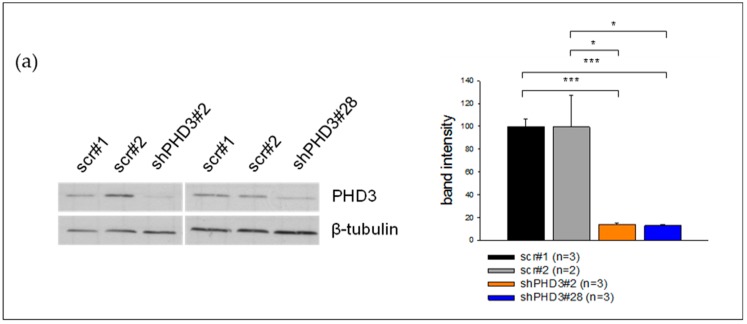

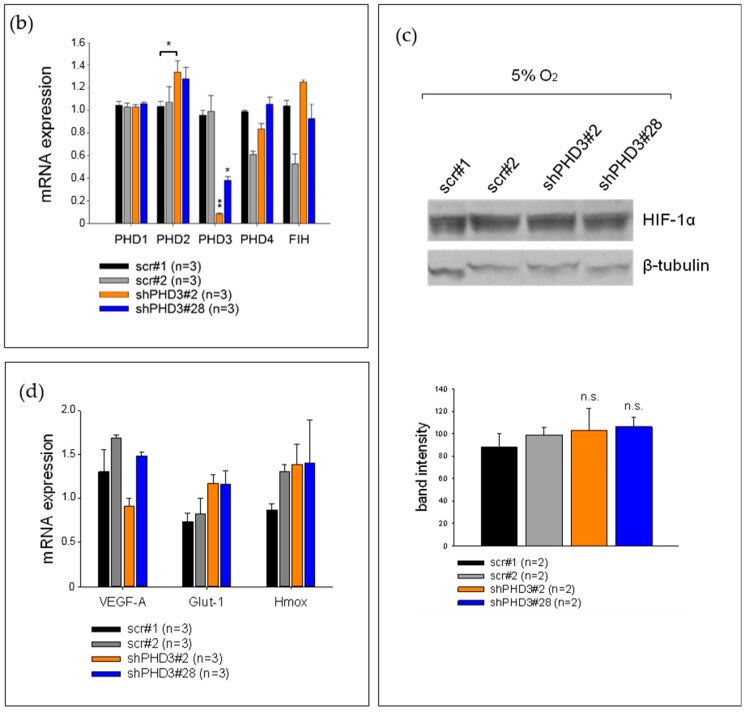
Down-regulation of PHD3 neither influences the HIF-1α protein level nor the expression of different HIF target genes in LM8 osteosarcoma cells. The levels of other prolyl hydroxylases are unchanged. (**a**) LM8 cells were transduced with lentiviral particles which delivered shRNA directed against PHD3 or a non-targeting (scr) shRNA. Immunoblotting reveals the PHD3 levels in the selected control and shPHD3 clones. β-tubulin was used as the loading control. The band intensities of three independent Western blots were quantified. A representative blot is shown. The error bars represent mean values ± SEM. (* *p* < 0.05, *** *p* < 0.001). (**b**) Depicted are relative mRNA expression levels of HIF-regulating enzymes (* *p* < 0.05, ** *p* < 0.01). (**c**) ShPHD3 clones were grown in an atmosphere of 5% oxygen (mild hypoxia) for 6 hours. Subsequently, they were lysed, and Western blotting was performed to detect HIF-1α. The band intensities of two independent Western blots were quantified. A representative blot is depicted. (**d**) qRT-PCR results of cell lysates (cells grown under normoxic conditions) show the relative mRNA expression of HIF-target genes (* *p* < 0.05, ** *p* < 0.01, *** *p* < 0.001).

We studied the role of PHD3 during tumor progression and angiogenesis by silencing its expression in murine LM8 osteosarcoma cells. Two independent shPHD3 RNAs were selected, and stable LM8 knock-down clones (shPHD3#2 and shPHD3#28) as well as control clones expressing a scrambled shRNA (scr#1 and scr#2) were established by lentiviral transduction. PHD3 protein and mRNA expression levels were found to be significantly reduced in shPHD3#2 and shPHD3#28 compared to control clones (Figure 1a,b). In order to evaluate whether PHD3 down-regulation leads to the stabilization of HIF-1α, Western blot and qRT-PCR analyses of cell lysates were conducted. We did not observe increased HIF-1α protein levels (Figure 1c; Supplementary Figure S1) or elevated HIF-target gene expression (Figure 1d). Quantification of the mRNA levels of PHD1, PHD2, PHD4 and FIH indicates that only PHD2 is mildly up-regulated in both shPHD3 clones (Figure 1b). HIF-2α is hardly detectable by Western blot analysis in LM8 cells even under conditions of severe hypoxia (1% oxygen for 24 hours, not shown). 

### 2.2. Reduction of PHD3 Leads to Accelerated Tumor Growth and Enlargement of Tumor Vessels

**Figure 2:**
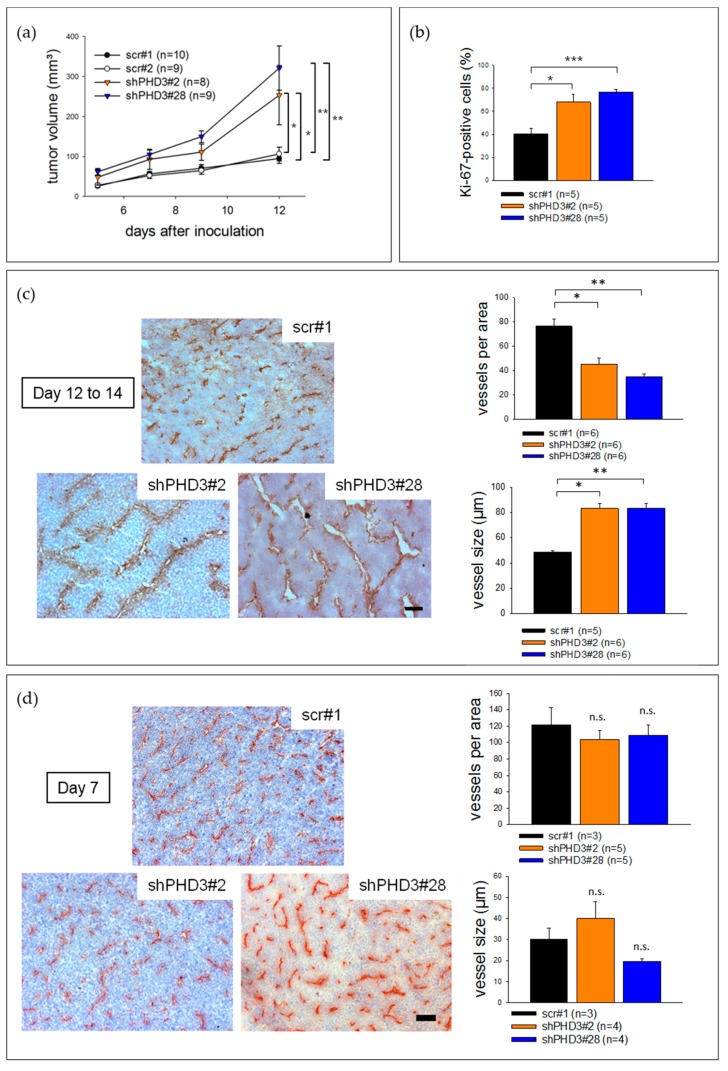
PHD3 silencing leads to accelerated tumor growth, reduced tumor vessel density and enlargement of tumor vessels. (**a**) PHD3-deficient LM8 or control clones were injected subcutaneously into C3H mice. Tumor size measurement was performed every 2 to 3 days. The error bars represent mean values ± SEM. (* *p* < 0.05, ** *p* < 0.01). (**b**) Tumor sections were stained for Ki-67 and with dapi. The number of Ki-67-positive nuclei was determined. (* *p* < 0.05, *** *p* < 0.001). (**c**) PECAM and hematoxylin staining was performed on tumor sections. To determine the vessel density, the number of vessels in a given area was counted. The vessel size was also measured. The error bars represent mean values ± SEM. (* *p* < 0.05, ** *p* < 0.01). Scale bar: 150 µm. (**d**) ShPHD3 and control clones were injected subcutaneously into mice and the tumor size was measured. The experiment was terminated at day 7 after inoculation. To characterize the tumor vessels, PECAM and hematoxylin staining on tumor sections was performed. The error bars represent mean values ± SEM. Scale bar: 150 µm.

In vitro proliferation assays of control and shPHD3 clones demonstrate that silencing of PHD3 reduces cell growth in culture (Supplementary Figure S2). To study the role of PHD3 during tumor growth we injected the shPHD3 clones #2 and #28 or control clones (scr#1, scr#2) subcutaneously into the flanks of C3H mice. Because our analyses revealed very similar in vivo and in vitro characteristics of LM8 clones scr#1 and scr#2 (Figure 1, Figure 2a), we chose to not further include scr#2 in the following analyses. The size of shPHD3 tumors was significantly increased as compared to control tumors (Figure 2a). Tumor-bearing mice were sacrificed and the tumors were isolated 12 to 14 days after inoculation. Ki-67 staining showed that shPHD3 tumors contain around twice as many proliferating cells as scr tumors (Figure 2b, Supplementary Figure S3). However, histological examination revealed no difference in the percentage of necrotic area in tumors with reduced PHD3 expression when compared to control tumors (data not shown). Immunohistochemistry staining for the endothelial cell marker PECAM (CD31) was performed to analyze vessel density and structure. We found that shPHD3 tumors contain less but larger vessels compared to their control counterparts (Figure 2c), suggesting that angiogenic sprouting is reduced.

**Figure 3:**
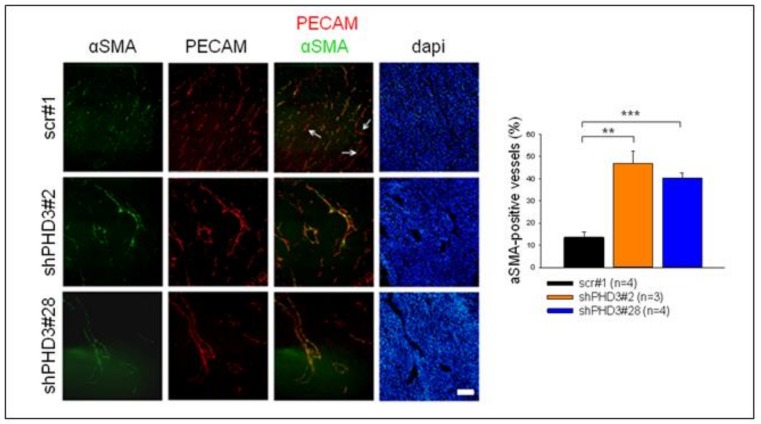
Tumor sections were co-stained for αSMA and PECAM, and the number of vessels covered with perivascular cells was counted. (** *p* < 0.01, *** *p* < 0.001). Scale bar: 100 µm.

**Figure 4:**
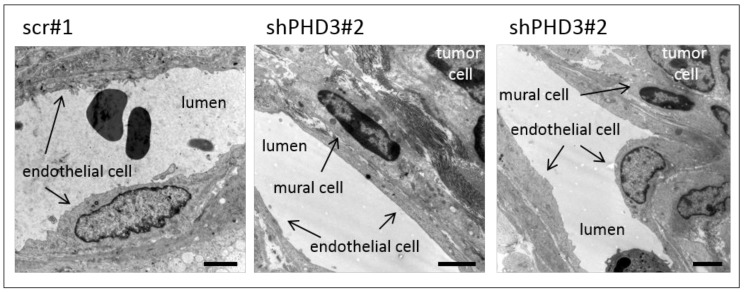
Blood vessels in shPHD3 tumors are lined with endothelium and contain perivascular cells. Pieces of tumor tissue were fixed, sectioned, and transmission electron micrographs of tumor blood vessels were taken. Control as well as shPHD3 tumor vessels are lined by endothelial cells. Vessels in shPHD3 tumors frequently contain perivascular mural cells. Scale bars: 2.5 µm.

Next, we investigated whether the changes in vessel morphology and perivascular cell coverage already occurred at the early stages of shPHD3 tumor growth. Tumors were prepared at day 7 post injection, and sections were stained for PECAM. The density and size of vessels were measured, and both were found to be similar in shPHD3 and control tumors (Figure 2d). Thus, angiogenesis is not altered in early-stage tumor development. In order to further characterize the vessel phenotype, tumor sections from 14-day-old tumors were stained for the perivascular marker αSMA and we observed that shPHD3 tumor vessels recruit perivascular cells more efficiently (Figure 3). Other pericyte markers, like NG2 or desmin, were not detected in LM8 tumors 2 weeks after inoculation (data not shown). αSMA staining of tumor sections that were prepared on day 7 after inoculation revealed that only very few vessels in all tumor groups are covered by perivascular cells and that the proportion of αSMA-positive vessels is not different between the control and shPHD3 tumors (data not shown). Electron micrographs show that the vessels in control tumors and in shPHD3 tumors (day 14) are lined by endothelial cells (Figure 4). Additionally, perivascular cells with pericyte-like appearance were observed in LM8 shPHD3 tumors. To address the question whether vessel function is altered, we examined the perfusion of vessels by injection of FITC-conjugated *Lycopersicon esculentum* lectin. The shPHD3 or control tumor vessels are equally well perfused (Supplementary Figure S4). Perfusion experiments using Dextran-TexasRed showed that shPHD3 tumor vessels have the tendency to be more leaky (Supplementary Figure S5). However, the difference is significant only for LM8 tumors derived from clone shPHD3#28 but not from clone shPHD3#2. Analysis of shPHD3#28 tumors and control tumors by hypoxyprobe staining showed that PHD3-deficient tumors contain significantly more and larger hypoxic areas than scr#1 tumors (Supplementary Figure S6). qRT-PCR experiments with tumor tissue samples did not reveal differences in the expression of HIF-target genes (Supplementary Figure S7).

### 2.3. PDGF-C Signaling Is Strongly Increased in LM8-shPHD3 Cells and Tumors

**Figure 5:**
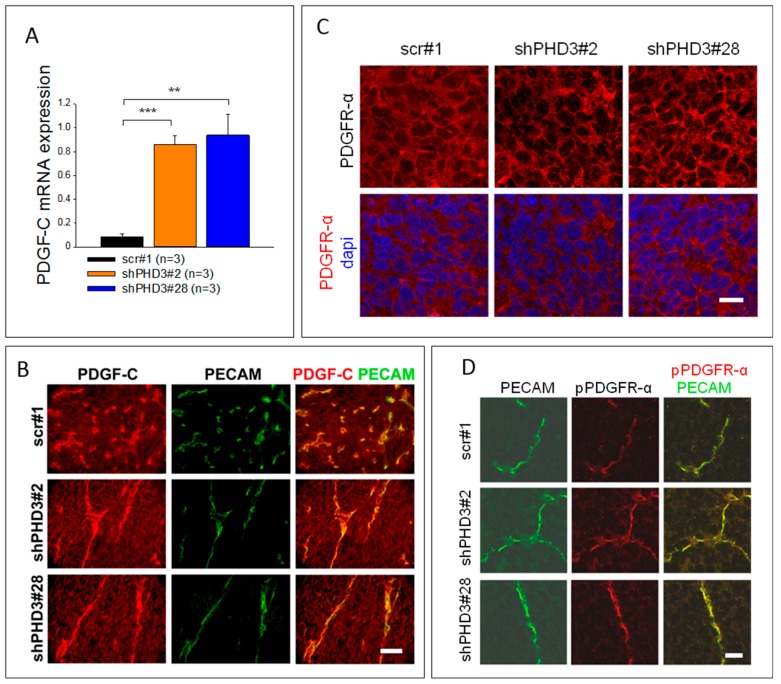
The expression of PDGF-C is strongly increased in shPHD3 tumors. PDGFR-α is localized at the plasma membrane of all cells in control and shPHD3 tumors. (**A**) In order to determine the PDGF-C expression level in PHD3 silenced tumors, qRT-PCR analyses of whole tumor lysates were performed. The error bars represent mean values ± SEM. (** *p* < 0.01, *** *p* < 0.001). (**B**) Tumor sections were stained to detect PDGF-C and PECAM. Scale bar: 50 µm. (**C**) Detection of PDGFR-α positive cells on tumor sections. Scale bar: 20 µm. (**D**) Co-immunofluorescence staining for PECAM and phosphorylated PDGFR-α (pPDGFR-α; pTyr754) shows receptor activation in endothelial cells. Scale bar: 25 µm.

Our observations of accelerated growth of shPHD3 tumors and the effect on tumor angiogenesis led us to search for the mediator of this phenotype. Various members of the PDGF family are known to be involved in tumor progression and angiogenesis [35,36,37,38,39]. Therefore, we determined the mRNA expression of PDGF family members. PDGF-A and -B are not differentially expressed (data not shown). However, PDGF-C expression is up-regulated in shPHD3 cells (Figure 5A, Supplementary Figure S8). Co-immunofluorescence staining of PDGF-C and PECAM on shPHD3 and control tumor sections showed that the tumor vessels strongly express PDGF-C (Figure 5B). The staining intensity on the tumor cells is weaker than in endothelial cells but shPHD3 tumors show stronger staining than control tumors. Next, we performed immunofluorescence staining of PDGFR-α on tumor sections. We found that PDGFR-α is expressed at the cell surface of virtually all cells in the different tumor groups (Figure 5C). Co-immunofluorescence staining of phosphorylated PDGFR-α (pTyr754) and PECAM showed that the receptor is activated on endothelial cells (Figure 5D).

### 2.4. Knock-Down of PDGF-C in shPHD3 Tumors Reduces Tumor Growth

**Figure 6:**
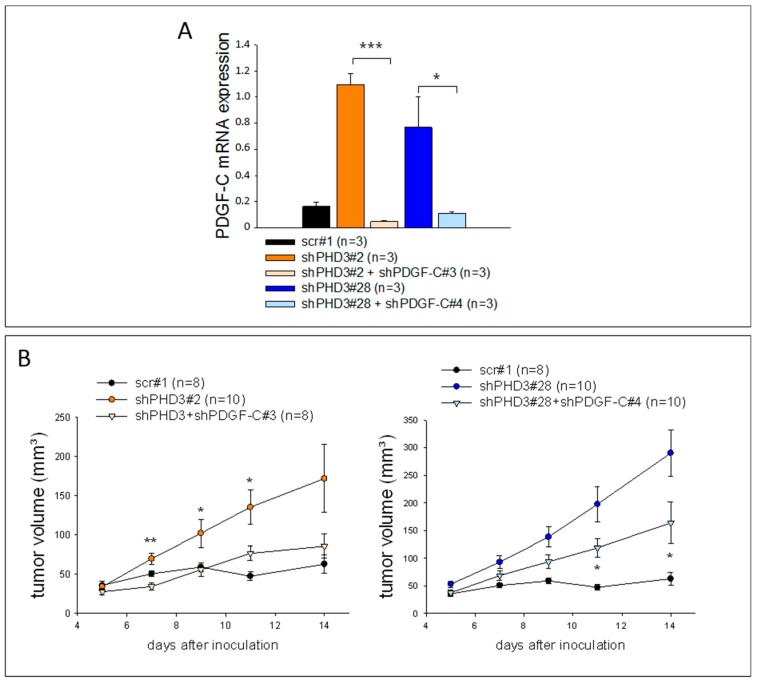
PDGF-C silencing decreases the growth of PHD3-deficient tumors. (**A**) Reduced PDGF-C mRNA expression in cultured shPHD3 LM8 cells transduced with lentiviral particles containing shRNA against PDGF-C. The PDGF-C expression levels were detected by qRT-PCR. The error bars represent mean values ± SEM. (* *p* < 0.05, *** *p* < 0.001). (**B**) Control, shPHD3 and shPHD3+shPDGF-C cell clones were inoculated subcutaneously and the tumor growth was measured. The error bars represent mean values ± SEM. Significant differences between shPHD3 and shPHD3+shPDGF-C tumors are indicated. (* *p* < 0.05, ** *p* < 0.01).

In order to evaluate whether PDGF-C indeed regulates tumor growth and angiogenesis in shPHD3 LM8 tumors, shPHD3 cells were transduced with lentiviral particles delivering shRNA against PDGF-C. qRT-PCR analysis of cell lysates of two selected clones, shPHD3#2+shPDGF-C#3 and shPHD3#28+shPDGF-C#4, reveals efficient PDGF-C silencing in vitro (Figure 6A). The PDGF-C knock-down cells also maintained the reduced PHD3 expression (Supplementary Figure S9). Subcutaneous injection of the double knock-down clones and the shPHD3 clones #2 and #28 demonstrated that shPHD3#2+shPDGF-C#3 and shPHD3#28+shPDGF-C#4 tumors grew significantly slower than tumors in which only PHD3 was silenced (Figure 6B) and that they contain fewer proliferating cells (Supplementary Figure S10). Reduced PDGF-C expression was confirmed in the respective tumors (Supplementary Figure S11).

### 2.5. The shPHD3 Vessel Phenotype Is Rescued in shPHD3+shPDGF-C Tumors

**Figure 7:**
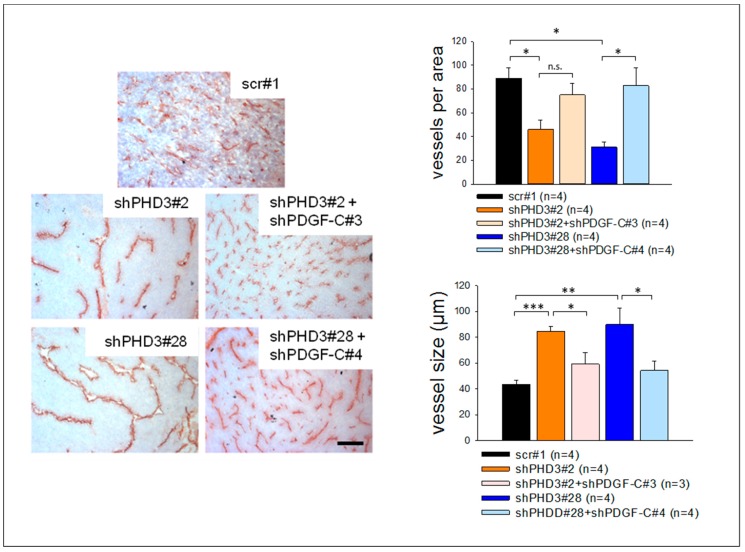
PDGF-C silencing reverses the shPHD3 tumor vessel phenotype. Sections from scr#1, shPHD3 and shPHD3+shPDGF-C tumors were stained immunohistochemically for PECAM and with hematoxylin. The vessel density and the vessel size were determined. The error bars represent mean values ± SEM. (* *p* < 0.05, ** *p* < 0.01, *** *p* < 0.001). Scale bar: 250 µm.

Tumor sections were stained to determine the vessel density, size and morphology in double knock-down tumors. Density and size of vessels in shPHD3+shPDGF-C tumors are comparable to those in control scr#1 tumors and are significantly reduced when compared to shPHD3 tumors (Figure 7). The percentage of αSMA-positive vessels in shPHD3+shPDGF-C double knock-down tumors is even lower than in scr tumors (Supplementary Figure S12), showing that PDGF-C is responsible for the increased perivascular cell coverage of shPHD3 tumor vessels. We also observed that down-regulating PDGF-C expression in PHD3 silenced tumors decreases the percentage of hypoxic tumor area (data not shown).

## 3. Discussion

The importance of hypoxia in the pathophysiology of tumors raises the question after the role of oxygen-sensing enzymes that control the response of cancer cells to hypoxia. Different studies showed that the cellular oxygen sensor, PHD2, restricts angiogenesis in experimental tumors, but can inhibit or enhance tumor growth in a tumor type-dependent manner [17,30,40]. Relatively little is known about the specific functions of other PHD family members. Here we corroborate the results of former studies showing that PHD3 acts as a tumor suppressor. Its inhibition in experimental mouse osteosarcoma led to accelerated tumor growth. Paradoxically, tumor growth stimulation was associated with a significant decrease in vessel density. Moreover, as a consequence of PHD3 inhibition, the tumor vasculature underwent dramatic phenotypic changes leading to the formation of large, irregularly-shaped vessels that are covered extensively by α-SMA positive perivascular cells. Analysis of the signaling pathways underlying the phenotypic changes shows that PHD3 deficiency does not enhance HIF-1α or VEGF-A expression in vitro or in vivo (Figure 1c,d, Supplementary Figures S1 and S7), but surprisingly we see upregulation of PDGF-C expression, which is involved in perivascular cell recruitment, vessel enlargement and growth stimulation.

In colorectal and gastric cancer as well as in glioma and astrocytoma PHD3 was also reported to function as a tumor suppressor [27,41,42,43]. In contrast, strongly tumor type-dependent effects on progression were reported for the related PHD2 protein. PHD2 inhibition promoted growth of experimental colon and pancreatic cancer [17], but blocked LM8 osteosarcoma and LLC carcinoma growth [30]. These results highlight the functional differences between different members of the PHD family, and show that specific functions of PHDs can differ dramatically depending on the tumor type.

The discrepancy between slower proliferation of shPHD3 tumor cells in vitro and faster growth rates in vivo suggests a pivotal contribution of the tumor microenvironment in the growth acceleration of PHD3-deficient tumors. One of the key processes influencing tumor progression is angiogenesis. However, vessel density was not altered in early stage tumors (Figure 4), at time points when the size of shPHD3 LM8 tumors was not significantly increased compared to control tumors, showing that the effect of PHD3 silencing manifests only later during tumor development. At day 12 or 14 after tumor cell inoculation, the (fast growing) PHD3-deficient tumors displayed fewer and enlarged vessels as compared to control tumors (Figure 2c). One possible explanation for the paradoxical finding—that shPHD3 tumors grow faster despite a strong decrease in vessel density—might be that the vasculature of PHD3-deficient tumors supplies the tumor tissue more efficiently with oxygen and nutrients. This is, however, unlikely to be the case, because lectin injection experiments in tumor-bearing mice failed to reveal differences in vessel perfusion between PHD3 knock-down tumors and control LM8 tumors (Supplementary Figure S4). Moreover, hypoxyprobe analysis showed that PHD3-deficient tumors have larger hypoxic areas than control tumors (Supplementary Figure S6), which is likely to be a consequence of the alterations of the tumor vasculature rather than a direct effect of the PHD3 silencing in the tumor cells. Hypoxia in tumor tissue is well known to promote aggressive tumor progression. Therefore, HIF-dependent or -independent effects of tumor hypoxia might contribute to the increased tumor growth.

In addition to affecting vessel density, shape and size, PHD3 has also an influence on the association of vessels with mural cells. PECAM-positive vessels in shPHD3 tumors are frequently covered with αSMA-positive cells. The exact identity of these mural cells is currently unclear, however, the lack of capillary pericyte markers, such as desmin or NG2, and their association with relatively large vessels indicates that these are vascular smooth muscle cells. Increased recruitment of mural cells in other tumor models was reported to lead to reduced vessel leakage [38,44], improved vessel integrity and functionality, and enhanced blood vessel growth [45] and tumor progression [46,47]. However, increased mural cell association in PHD3-deficient tumors apparently does not improve vessel function, but might be necessary for stabilizing the abnormally enlarged vessels. It was reported a decade ago that tumor endothelial cells can undergo endothelial-to-mesenchymal transition (EndMT), leading to the emergence of PECAM/αSMA double positive cells [48]. The co-localization of PECAM and αSMA observed in the vasculature of PHD3-deficient tumors is consistent with the possibility that endothelial cells underwent EndMT.

In tumors of PHD3-deficient LM8 cells, silencing of PDGF-C expression reduces tumor growth (Figure 6B); the tumor vessels are smaller, more numerous, and contain fewer mural cells (Figure 7 and Supplementary Figure S12). These results suggest that PDGF-C upregulation in PHD3-deficient tumor cells contributes significantly to the observed changes in tumor vasculature and increased tumor growth. In light of the fact that PDGF-C was up-regulated already in cultured tumor cells, its activity in tumors seems to be a cause, rather than a consequence of increased tumor growth. Members of the PDGF family can promote tumor progression in several ways. PDGF-C stimulates the recruitment of perivascular cells [38,49,50], endothelial cell migration, mobilization of endothelial progenitor cells [51], and other processes during tumor progression [35,37,38,49,50,52]. To address the question of which cells are activated by PDGF-C signaling in LM8-shPHD3 tumors, we analyzed the expression and activation pattern of its cognate receptor, PDGFR-α. This transmembrane protein is expressed by virtually all cells in control and in shPHD3 tumors. PDGFR-α phosphorylated on Tyr754 was co-localized with PECAM (Figure 5D), suggesting that PDGFR-α signaling occurs preferentially in endothelial cells. A direct effect of PDGF-C on endothelial cells was also reported by several former studies [50,51,53]. A possible explanation for the preferential activation of PDGFR-α in the cells that line blood vessels might be that the proteases that cleave the latent PDGF-C pro-form [36,52,54,55,56,57,58] are provided by the blood stream or by endothelial cells themselves [59,60].

However, PDGF-C might act also on non-endothelial cells, including tumor cells and perivascular cells, because (i) autocrine growth stimulation of tumor cells by PDGF-C was not observed under cell culture conditions but might occur in the tumors; (ii) PDGFR-α can be phosphorylated and thereby activated on various additional tyrosine residues [61] that are not detected via staining with the PDGFR-α (Tyr754)-specific antibody used in our study; and (iii) PDGF-C was proposed to bind also to receptors other than PDGFR-α [62]. It is therefore reasonable to assume that PDGF-C exerts multiple effects on various cell types in PHD3-deficient tumors, which altogether induce the phenotypic changes of the tumor vasculature and promote tumor growth.

Interestingly, we have previously observed that overexpression of FIH in LM8 tumors also elevates PDGF-C expression and tumor growth [38], showing that the PDGF-C pathway is influenced in LM8 tumors by at least two different oxygen sensors in opposite ways. However, in the case of FIH overexpression, enhanced PDGF-C signaling increases the maturation of the tumor vasculature, without influencing vessel density or vessel size. Thus, the vessel phenotypes in PHD3-deficient and FIH-overexpressing LM8 tumors are different. It is conceivable that the increased FIH expression creates conditions unequal to those caused by PHD3 silencing such that the consequences of elevated PDGF-C levels are not identical in both models. While our findings provide evidence for a novel link between PHD3 and the PDGF-C signaling pathway, the molecular interactions involved here remain to be elucidated. Our preliminary analyses indicate that the NF-κB pathway which can be influenced by PHD3 [27] is not responsible for the PDGF-C expression in this context (data not shown). PDGF-C was shown to be a target of HIF-1α-mediated transcriptional activation [63], and small changes in HIF-1α expression which are not readily detected by Western Blot analyses could still be involved in the up-regulation of PDGF-C in PHD3-deficient LM8 cells and tumors, especially if one takes into account that tumor hypoxia was increased (Supplementary Figure S6).

The cross-talk between the PHD3 and PDGF-C pathways is most probably tumor type-specific because we have been unable to detect up-regulation of PDGF-C after silencing of PHD3 in LLC cells (data not shown). This would not be without precedence, for example, inhibition of PHD2 in human colon carcinoma cells promotes tumor cell proliferation via NF-kB signaling [17], whereas tumor growth is inhibited via TGF-β signaling in murine tumor cell lines such as LM8 [30,40]. Although we were unable to detect consistent changes in the expression of HIF-1 or HIF-2α in LM8 cells, we cannot exclude the possibility that subtle changes in their expression levels, induced by PHD3 deficiency, contributed to the phenotypic alterations observed in the respective experimental tumors. Clearly, the results obtained in the LM8 tumor model cannot be simply generalized. It is, however, important to note that the modulation of enzymes such as PHD3, which presumably affects the expression of many genes, may cause divergent effects, depending on the genetic alterations that have manifested in specific tumor types or even individual tumors.

## 4. Materials and Methods

### 4.1. Cell Culture and Virus Production

Murine LM8 osteosarcoma cells (kindly provided by Dr. C. Beltinger, Ulm, Germany) were cultured in MEM-alpha medium (Thermo Fisher Scientific, Darmstadt, Germany) supplemented with 10% fetal bovine serum, 1% non-essential amino acids and 1% L-glutamine in 5% CO_2_. Experiments under hypoxia were conducted as described earlier [30,38]. To evaluate tumor cell proliferation in vitro, 1 × 10^5^ cells were seeded in 6-well plates in three independent experiments. Cells were trypsinized after 24 or 48 hours and the cell number was counted using a CASY cell counter (Innovatis, Bielefeld, Germany). 293T cells were cultured in DMEM, glutaMAX™ medium (Thermo Fisher Scientific, Darmstadt, Germany) including 10% fetal bovine serum in atmosphere containing 7.5% CO2. In order to produce lentiviral particles (see paragraph below), 293T cells were transfected with the lentiviral vectors pLVTHM, psPAX2 and pMD2.G (kind donation of Dr. D. Trono, Geneva, Switzerland) using the transfection reagent polyethylenimine (1 mg/mL; Sigma-Aldrich, Munich, Germany). Lentiviral particles were harvested 48 hours after transfection.

### 4.2. Silencing of PHD3 and PDGF-C

Stable knock-down of PHD3 was achieved essentially as described [64,65]. LM8 cells were transduced with conditioned medium containing lentiviral particles which were taken from transfected 293T cells. The viral particles carried one of two independent shPHD3 RNAs or a non-targeting (scr) shRNA (Table 1) cloned into the pLVTHM vector. To knock down PDGF-C expression in shPHD3 LM8 clones, a specific PDGF-C shRNA (Table 1) was introduced via lentiviral transduction. Single shRNA-expressing cells were sorted per well into 96-well plates by FACS (BD FACSAria™ II; BD Biosciences, Heidelberg, Germany; excitation 488 nm, filters LP 502 and LP 530/30) on the basis of green fluorescence.

### 4.3. Tumor Model and Mice

In order to generate experimental tumors, 2 × 10^6^ cells were injected subcutaneously into both flanks of C3H mice (TU Dresden, Dresden, Germany). Five mice were used for each independent tumor experiment (10 tumors per experiment). Every 2 to 3 days the tumor size was measured and the volume was calculated using the formula (a^2^ × b)/2, where a is the minor tumor axis and b is the major tumor axis. If tumor cell injection did not lead to the development of a tumor or if cells had accidentally been injected into the musculature instead of subcutaneously, which happened very rarely, the respective number of analyzed tumors decreased, and is indicated as *n* = 8 or *n* = 9 (instead of *n* = 10). The tumor-bearing mice were sacrificed after 12 to 14 days if not otherwise stated. Then the tumors were isolated and embedded in Tissue-Tek^®^ OCT™ compound (TTEK, Sakura, Staufen, Germany). All animal experiments were approved by the State of Saxony (24-916811-1/2011-26, Landesdirektion Dresden) and were performed according to the guidelines of the ethical committee of the Faculty of Medicine, TU Dresden, Dresden, Germany.

### 4.4. RNA Extraction and qRT-PCR

The preparation of RNA was performed from cell lysates using the Universal RNA Purification Kit (Roboklon, Berlin, Germany). For extraction from whole tumor lysates, the RNeasy Mini Kit (Qiagen, Hilden, Germany) was used. One to two micrograms of RNA were transcribed with SuperScript^®^ II reverse transciptase (Thermo Fisher Scientific, Darmstadt, Germany). Ten nanograms of cDNA per reaction were subjected to quantitative PCR (qPCR) using the Maxima™ SYBR Green qPCR Master Mix (Fermentas GmbH, Leon-Rot, Germany) and the iCycler iQ (BIO-RAD Laboratories GmbH, Munich, Germany). *Tbp* and *Eef2* were used as reference genes. All primer sequences are listed in Table 2.

### 4.5. Western Blot

Immunoblot analysis was performed as described [38]. The following antibodies were used for protein detection: anti-PHD3 (NB100-303, Novus Biologicals, Littleton, CO, USA), anti-PDGF-C (AF-1447, R&D systems, Wiesbaden, Germany), anti-HIF-1α (10006421, Cayman Chemical, Michigan, MI, USA) and anti-β-tubulin (RB-9249-P1, Neomarkers, Fremont, CA, USA). The band intensities were quantified using the Quantity One analysis software (Bio-Rad Laboratories GmbH, Munich, Germany).

### 4.6. Immunostaining and Microscopy

Frozen 10 µm tumor tissue sections were prepared for all analyses except for determination of vessel leakiness and vessel perfusion (14 µm; see below). For immunohistochemistry analysis the sections were stained with an anti-PECAM antibody and co-stained with hematoxylin. In these sections, the number of vessels was counted in a non-necrotic area (890 µm × 670 µm) using the public domain NIH Image program (developed at the U.S. National Institutes of Health and available on the Internet at http://rsb.info.nih.gov/nih-image/) to calculate the vessel density. The vessel size was measured with the help of the Analysis^B^ software (Olympus Europa GmbH, Hamburg, Germany). Tumor sections were stained with anti-PDGFR-α (NB110-61020, Novus Biologicals, Littleton, CO, USA) or anti-pPDGFR-α antibody (sc-18228, Santa Cruz, Heidelberg, Germany). Perivascular cells were stained with an antibody detecting alpha-smooth muscle actin (αSMA; F3777, Sigma-Aldrich, Munich, Germany). Labeling with the proliferation marker Ki-67 was performed with an antibody from DAKO Diognostika GmbH (M7249, Hamburg, Germany). To examine stained sections, an Axioplan 2 microscope (Carl Zeiss, Munich, Germany) was used. The light micrographs were captured with the Axiocam MRc5 camera and the AxioVision AC Rel. 4.5 acquisition software (Carl Zeiss, Munich, Germany). Immunofluorescence images were taken with the help of a QImaging Retiga 2000R camera (QImaging, Surrey, BS, Canada) and the ImagePro MC6.0 software (Media Cybernetics, Inc., Rockville, MD 20850, USA).

For electron microscopic imaging, tissue samples were fixed in 2.5% glutaraldehyde in 0.1 M (pH 7.3) sodium cacodylate buffer for 3 days at 4 °C and then post-fixed in 2% osmium tetroxide solution for 1 hour. After fixation, the samples were dehydrated in a graded series of ethanol and then embedded in Epon resin. Ultrathin sections of 70 nm were cut and placed on Formvar-coated copper grids. The sections were counterstained with uranyl acetate and lead citrate and were viewed with a Zeiss EM 906 transmission electron microscope (Carl Zeiss, Munich, Germany) operated at 80 kV.

### 4.7. Determination of Hypoxic Area, Vessel Perfusion, Leakage and Tumor Necrosis

To detect hypoxic areas in the tumors we used the Hypoxyprobe–Omni kit (Hypoxyprobe Inc, Burlington, MA, USA). Sixty milligrams of hypoxyprobe per kilogram body weight were injected intraperitoneally into tumor-bearing mice. Two hours later the mice were sacrificed, and the tumors were collected. Staining of tumor sections was performed according to the manufacturers protocol. The vessel perfusion was tested by intravenous injection of 0.1 mg FITC-conjugated Lycopersicon esculentum lectin (VC-FL-1171-M001, Axxora Deutschland GmbH, Lörrach, Germany). After 10 minutes the mice were sacrificed, the heart was perfused with PBS and 2% PFA and the tumor tissue was frozen. For detection of vessel leakiness, dextran-TexasRed (D1864, Thermo Fisher Scientific, Darmstadt, Germany) was injected into the tail vein and the mice were heart perfused as described above. The necrotic areas in tumors were measured on hematoxylin-stained tumor sections. Hematoxylin positive areas were considered to be viable areas.

### 4.8. Statistics

The *t*-test was performed for statistical analysis of all experiments as described [38].

## 5. Conclusions

Taken together, our observations and those by others highlight the role of PHD3 as a tumor suppressor [27,41,42,43]. Moreover, we show for the first time that PHD3 inhibition in cancer cells has profound effects on the tumor vasculature by influencing vessel morphology and the emergence of mural cells in a PDGF-C-dependent manner. The phenotypic alterations observed in PHD3-deficient osteosarcoma are in line with the hypothesis that PHD3 limits PDGF-C expression in tumor cells, inhibits the recruitment of perivascular cells, and restricts tumor cell proliferation in a tumor cell non-autonomous fashion. Important insights into the specific functions of different PHDs come from our systematical comparison of the roles played by PHD2 [30,40] and PHD3 (this study), performed in the same tumor model. This analysis allows us to conclude that PHD2 and PHD3 can exert different, and in some respect even opposite functions during tumor progression. Further work is needed to delineate the mechanisms that balance the activity of the different PHDs in tumors and hence, control their action during tumor vascularization and growth.

## Figures and Tables

**Table 1:** shRNA oligonucleotides.

Clone	shRNA Sequence
shPHD3#2	**Sense**
	5’-CGCGTCCCGTCCAAGGCAATGGTGGCTTGTTCAAGAGACAAGCCAC CATTGCCTTGGACTTTTTGGAAAT-3’
	**Antisense**
	3’-AGGGGCAGGTTCCGTTACCACCGAACAAGTTCTCTGTTCGGTGGT AACGGAACCTGAAAAACCTTTAGC-5’
shPHD3#28	**Sense**
	5’-CGCGTCCCGCAAATACTATGTCAAGGATTCAAGAGATCCTTGACATA GTATTTGCTTTTTGGAAAT-3’
	**Antisense**
	3’-AGGGGCGTTTATGATACAGTTCCTAAGTTCTCTAGGAACTGTATCA TAAACGAAAAACCTTTAGC-5’
scr#1, scr#2	**Sense**
	5’-CGCGTCCCAGTCGCTTAGAAACGAGAATTCAAGAGATTCTCGTTTCT AAGCGACTTTTTTGGAAAT-3’
	**Antisense**
	3’-AGGGGTCAGCGAATCTTTGCTCTTAAGTTCTCTAAGAGCAAAGATT CGCTGAAAAAACCTTTAGC-5′
shPDGF-C#3, shPDGF-C#4	**Sense**
	5′-CGCGTCCCAGTGGTGAATCTGAATCTCTTCAAGAGAGAGATTCAGAT TCACCACTTTTTTGGAAAT-3′
	**Antisense**
	3′-AGGGGTCACCACTTAGACTTAGAGAAGTTCTCTCTCTAAG TCTAAGTGGTGAAAAAACCTTTAGC-5′

**Table 2:** qRT-PCR primer sequences.

Primer	Sequence
Eef2	5′-atc ctc acc gac atc acc aag-3′
	5′-ctg ctc tgg aca ctg gat ctc-3′
FIH	5′-gta ctg gtg gca cca tat ag-3′
	5′-cct ctc caa gca tct tct ca-3′
Glut-1	5′-gtc ggg ggc atg att ggt tcc tt-3′
	5′-ctc ttg gcc cgg ttc tcc tcg tta-3′
Hmox	5′-ttg tct gag gcc ttg aag ga-3′
	5′-ctg ctt gtt gcg ctc tat ct-3′
PDGF-A	5′-gag ata ccc cgg gag ttg at-3′
	5′-aaa tga ccg tcc tgg tct tg-3′
PDGF-B	5′-gat ctc tcg gaa cct cat cg-3′
	5′-ggc ttc ttt cgc aca atc tc-3′
PDGF-C	5′-gtg gag gaa att gtg cct gt-3′
	5′-tcc aga gcc aca tca gtg ag-3′
PDGFR-α	5′-cca cca gtg aag tct atg ag-3′
	5′-acg cat tat cag agt cca cc-3′
PHD1	5′-tct acc cag gca atc tgg tc-3′
	5′-gct agg ctg agg gag gaa gt-3′
PHD2	5′-cat acg cca caa ggt acg ca-3′
	5′-aac tga gag gct gta ggt ga-3′
PHD3	5′-ggc cgc tgt atc acc tgt at-3′
	5′-ttc tgc cct ttc ttc agc at-3′
PHD4	5′-acc tcc tgt cgc tac atg a-3′
	5′-cac agt gcc ttc gag tgt cc-3′
Tbp	5′-tct acc gtg aat ctt ggc tgt aaa-3′
	5′-ttc tca tga tga ctg cag caa a-3′
VEGF-A	5′-agt ccc atg aag tga tca agt tca-3′
	5′-atc cgc atg atc tgc atg-3′

## Supplementary Materials

The following are available online at http://www.mdpi.com/2072-6694/10/12/496/s1, Figure S1: HIF-1α protein levels are not altered in LM8 cells cultured in hypoxic conditions, Figure S2: In vitro proliferation assay of shPHD3 and control clones, Figure S3: PHD3-deficient tumors are more proliferative, Figure S4: Vessel perfusion in shPHD3 and control tumors, Figure S5: Vessel leakiness in shPHD3 and control tumors, Figure S6: ShPHD3#28 tumors are more hypoxic than control tumors, Figure S7: Silencing of PHD3 does not influence the VEGF-A or Glut-1 expression in LM8 tumors, Figure S8: PDGF-C is up-regulated by reduction of PHD3 in vitro, Figure S9: PHD3 expression in double knock down LM8 clones, Figure S10: ShPHD3+shPDGF-C double know-down tumors are less proliferative compared to tumors in which PHD3 was silenced only, Figure S11: PDGF-C is significantly reduced in shPHD3+shPDGF-C LM8 tumors, Figure S12: Knock down of PDGF-C in PHD3-deficient tumors reduces perivascular cell coverage of tumor vessels.
