# Supplementary material for: PHD3 Acts as Tumor Suppressor in Mouse Osteosarcoma and Influences Tumor Vascularization via PDGF-C Signaling
Source: Cancers (Basel). 2018 Dec 6;10(12):496. doi: 10.3390/cancers10120496 (PMC6316346; doi:10.3390/cancers10120496)
Supplement: Supplementary file 1 [file cancers-10-00496-s001.pdf]

Supplementary Material

PHD3 Acts as Tumor Suppressor in Mouse Osteosarcoma and Influences Tumor Vascularization via PDGF-C Signaling

Antje Egners, Maryam Rezaei, Aleksandar Kuzmanov, David M. Poitz, Doreen Streichert, Thomas Müller-Reichert, Ben Wielockx and Georg Breier

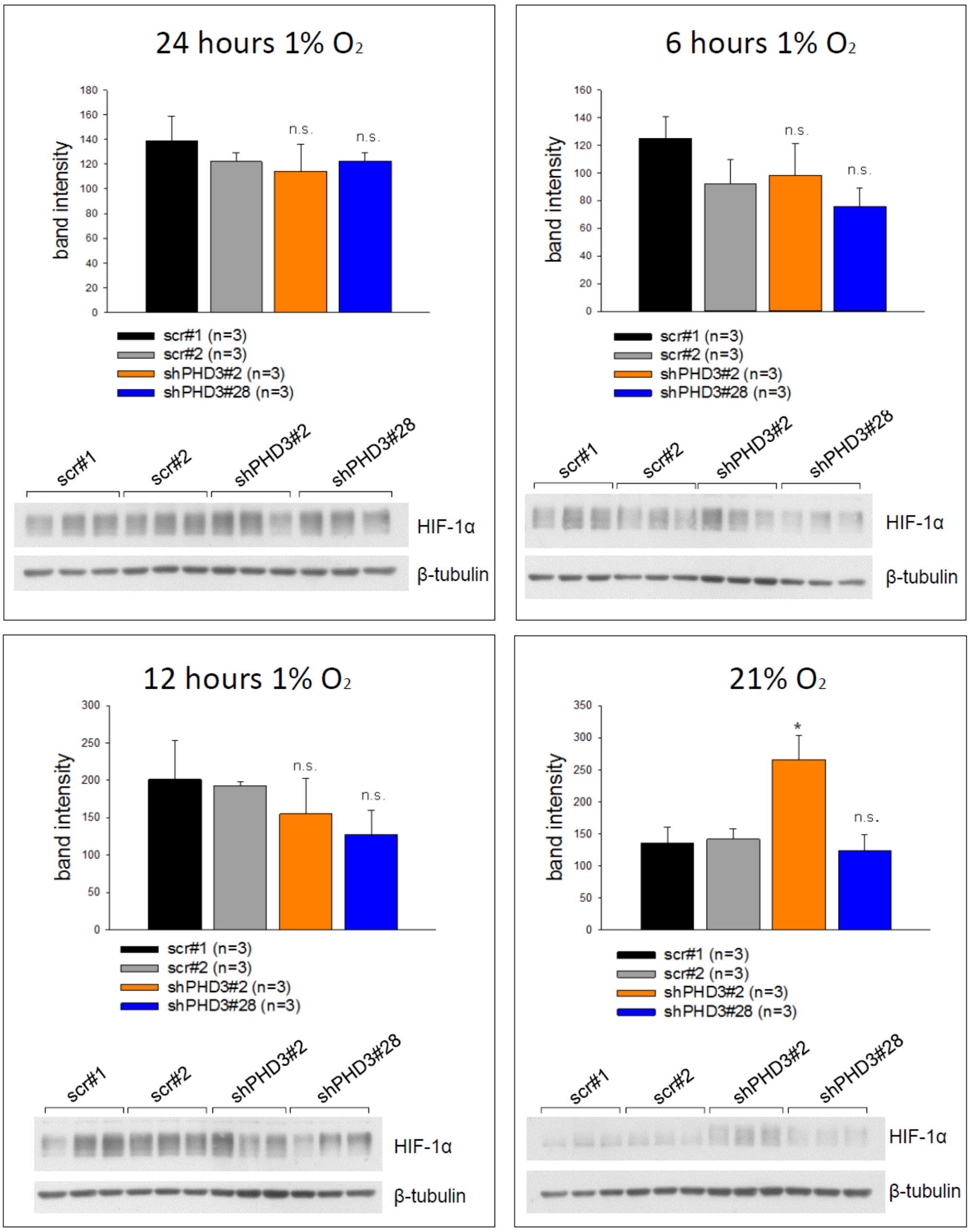

Figure S1. HIF-1α protein levels are not altered in LM8 cells cultured in hypoxic conditions.

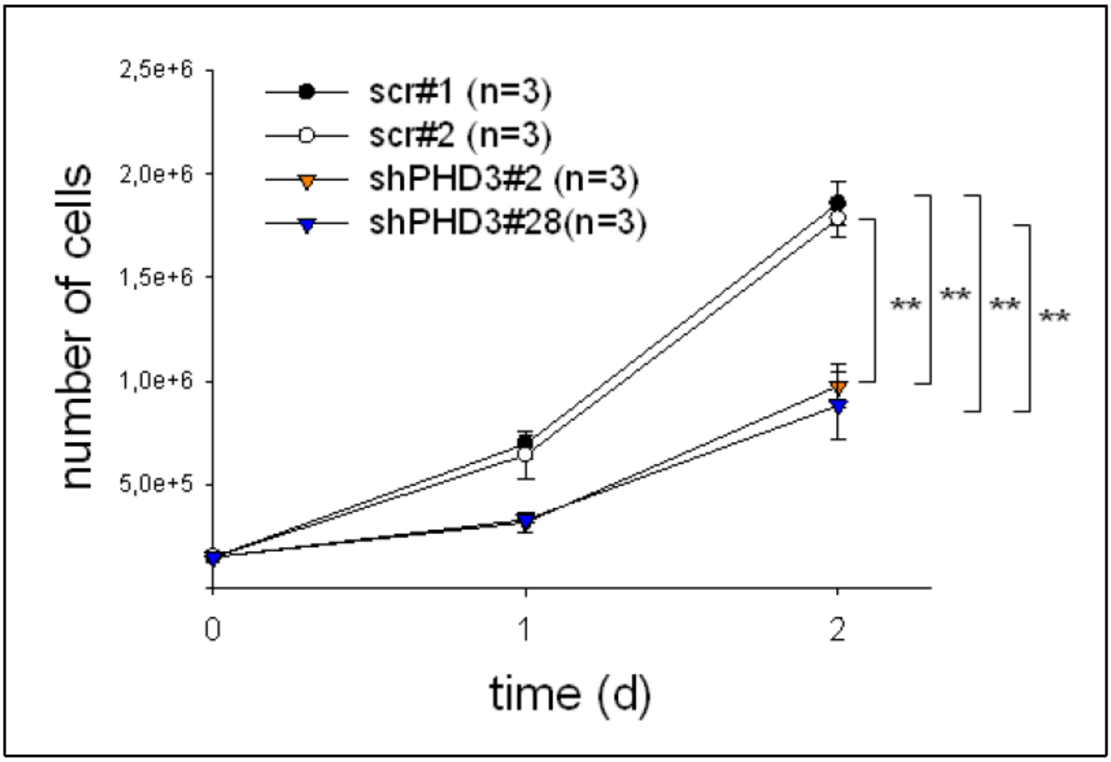

Figure S2. In vitro proliferation assay of shPHD3 and control clones.

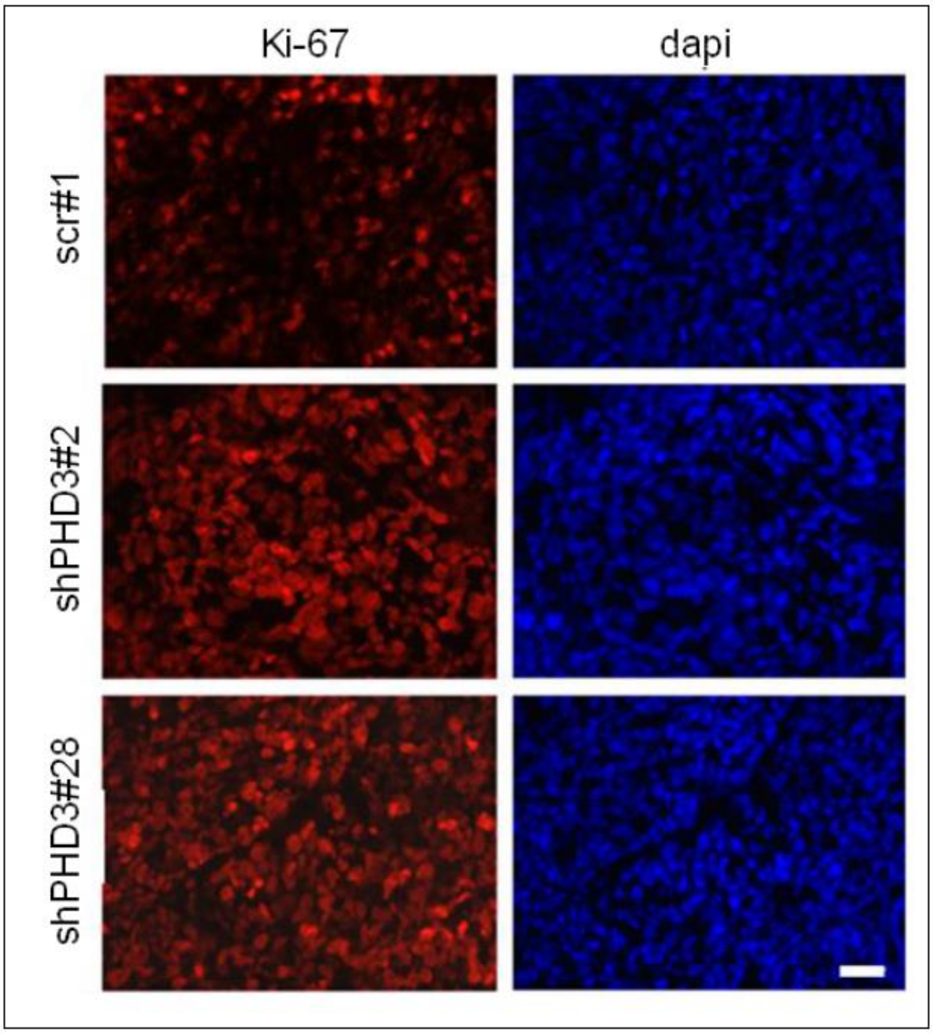

Figure S3. PHD3-deficient tumors are more proliferative.

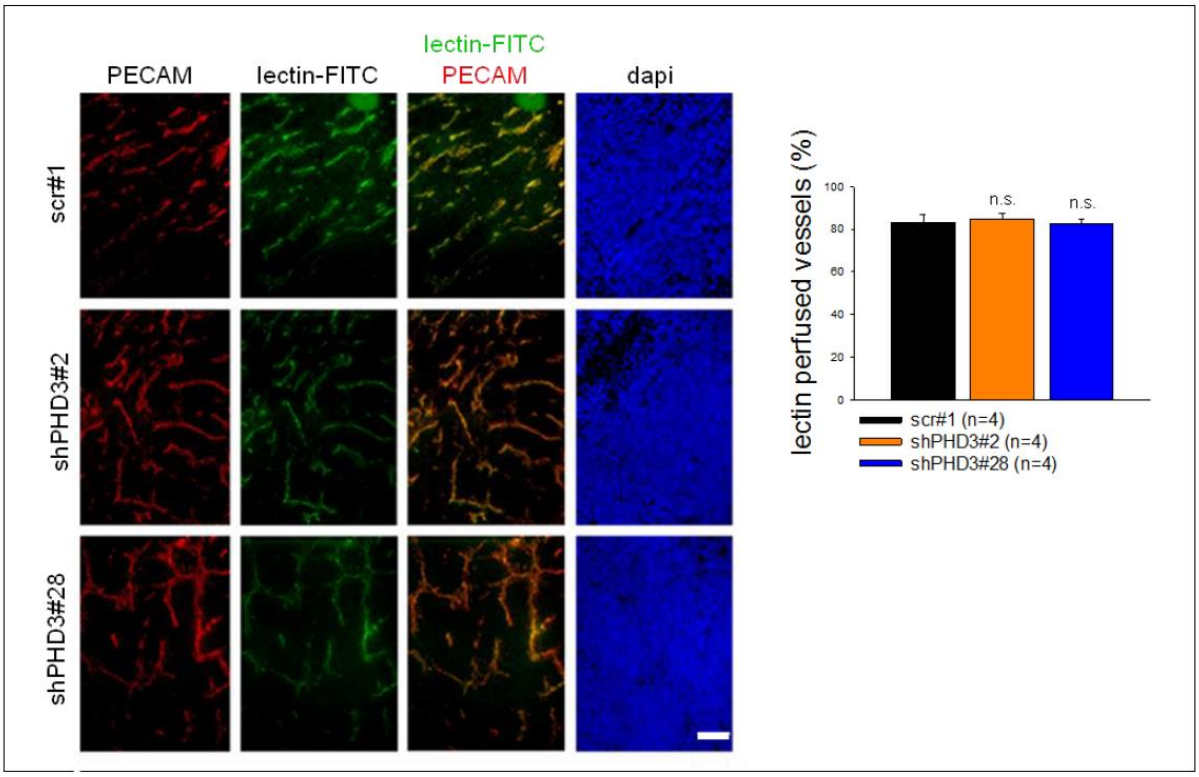

Figure S4. Vessel perfusion in shPHD3 and control tumors.

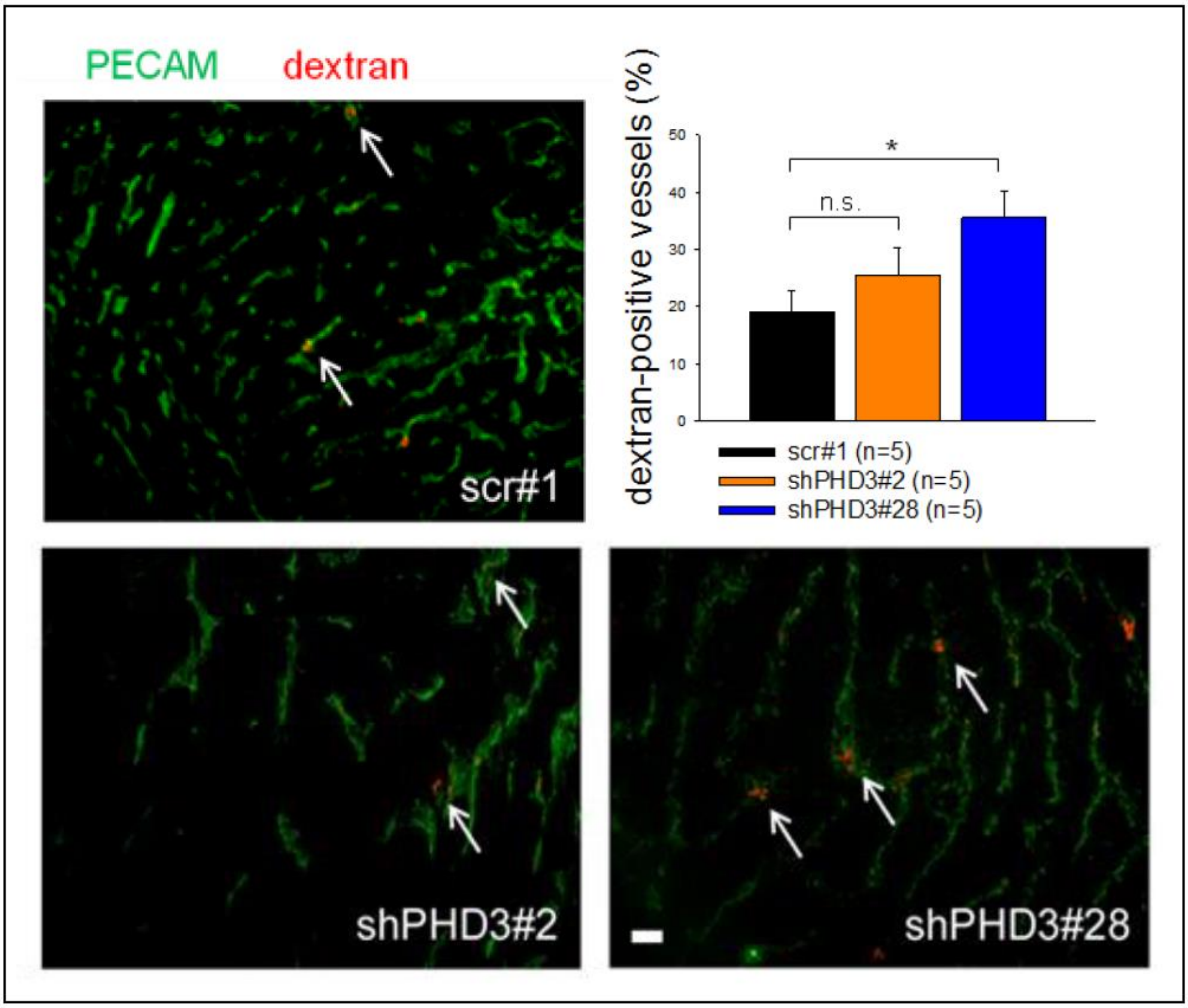

Figure S5. Vessel leakiness in shPHD3 and control tumors.

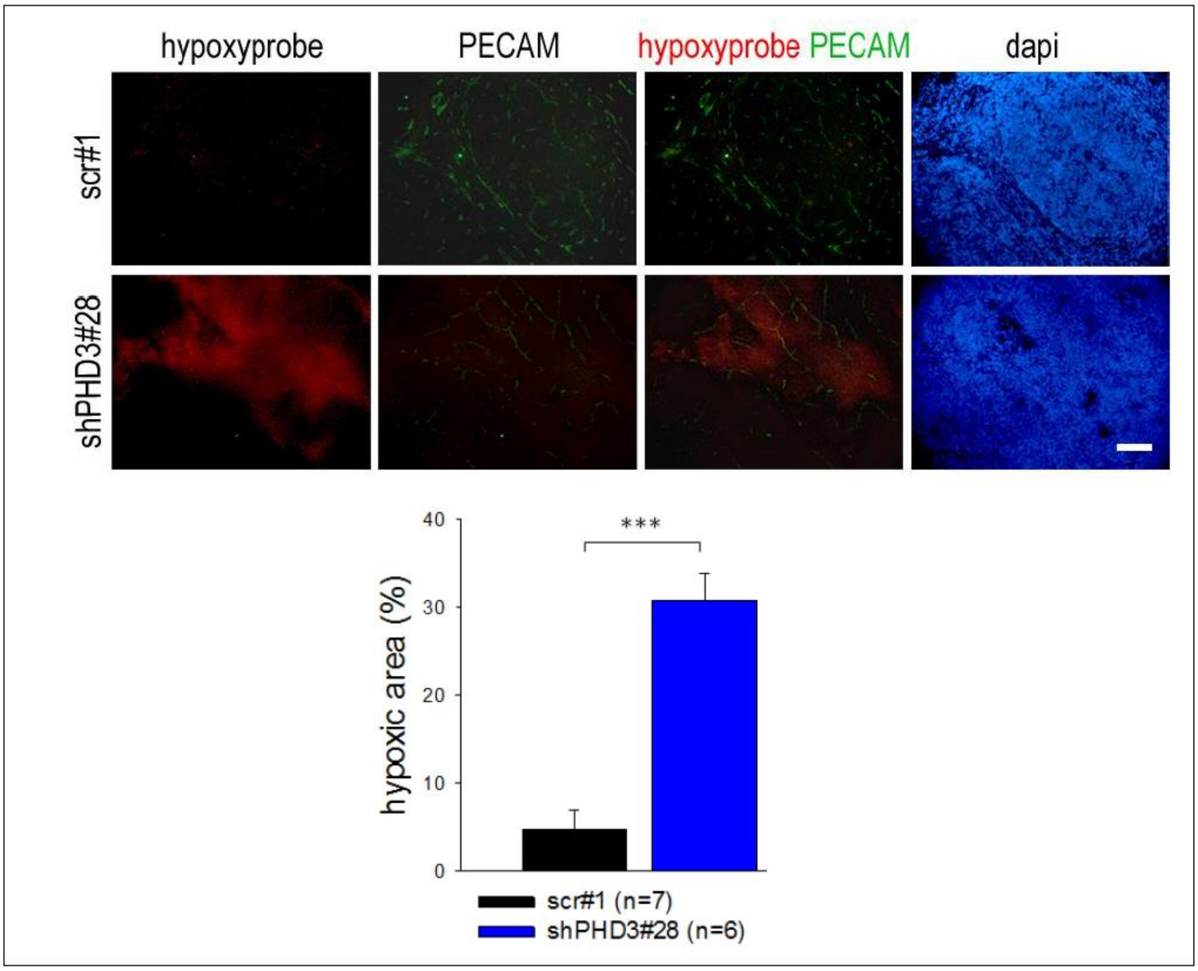

Figure S6. ShPHD3#28 tumors are more hypoxic than control tumors.

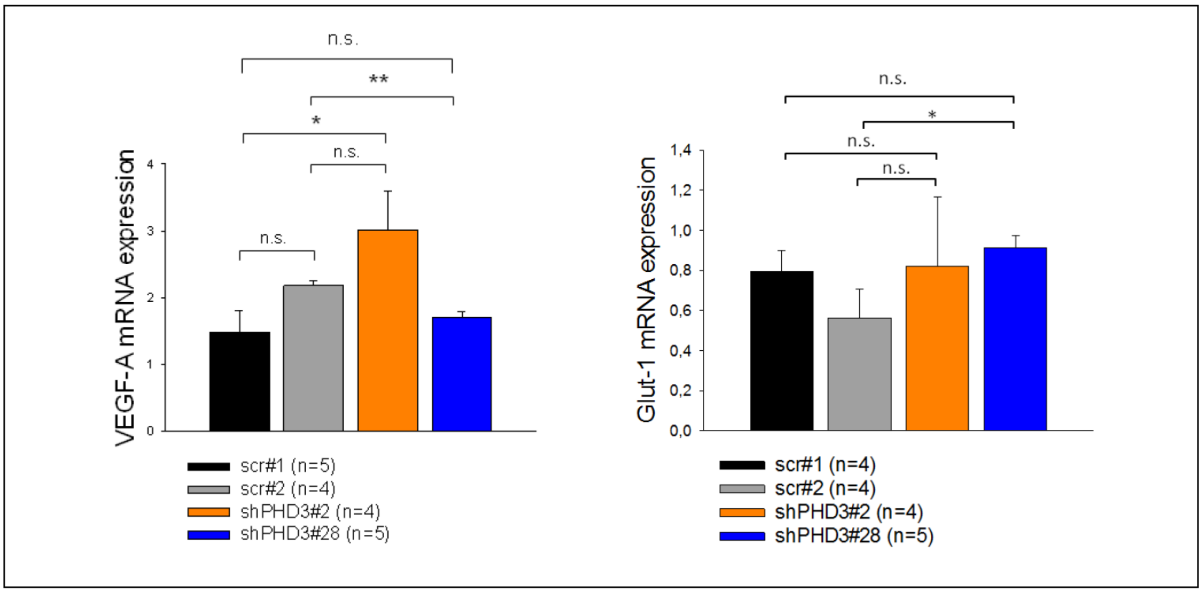

Figure S7. Silencing of PHD3 does not influence the VEGF-A or Glut-1 expression in LM8 tumors.

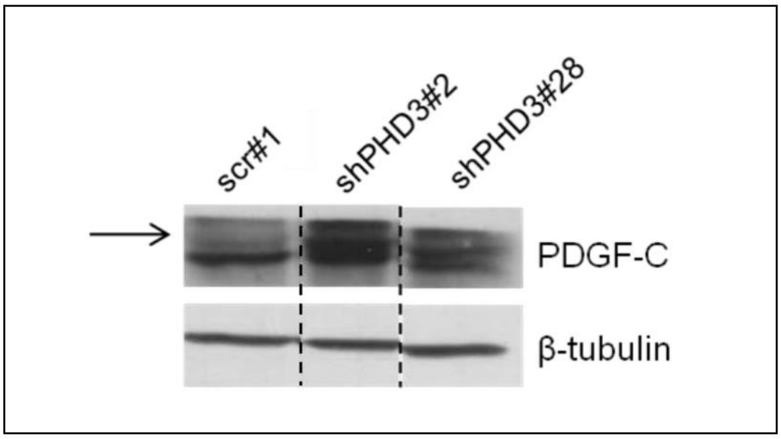

Figure S8. PDGF-C is up-regulated by reduction of PHD3 in vitro.

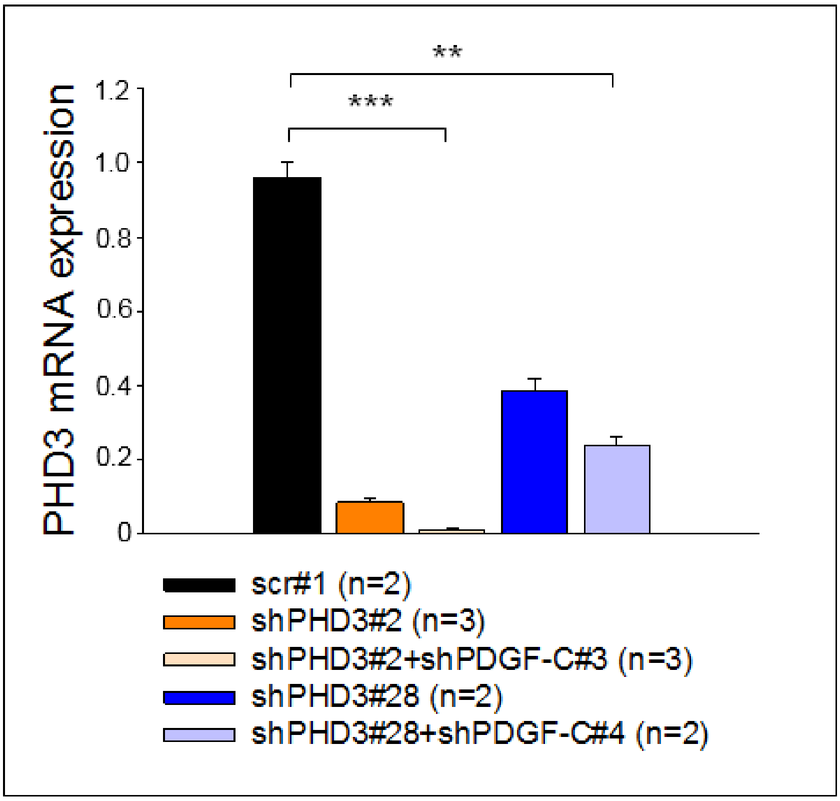

Figure S9. PHD3 expression in double knock down LM8 clones.

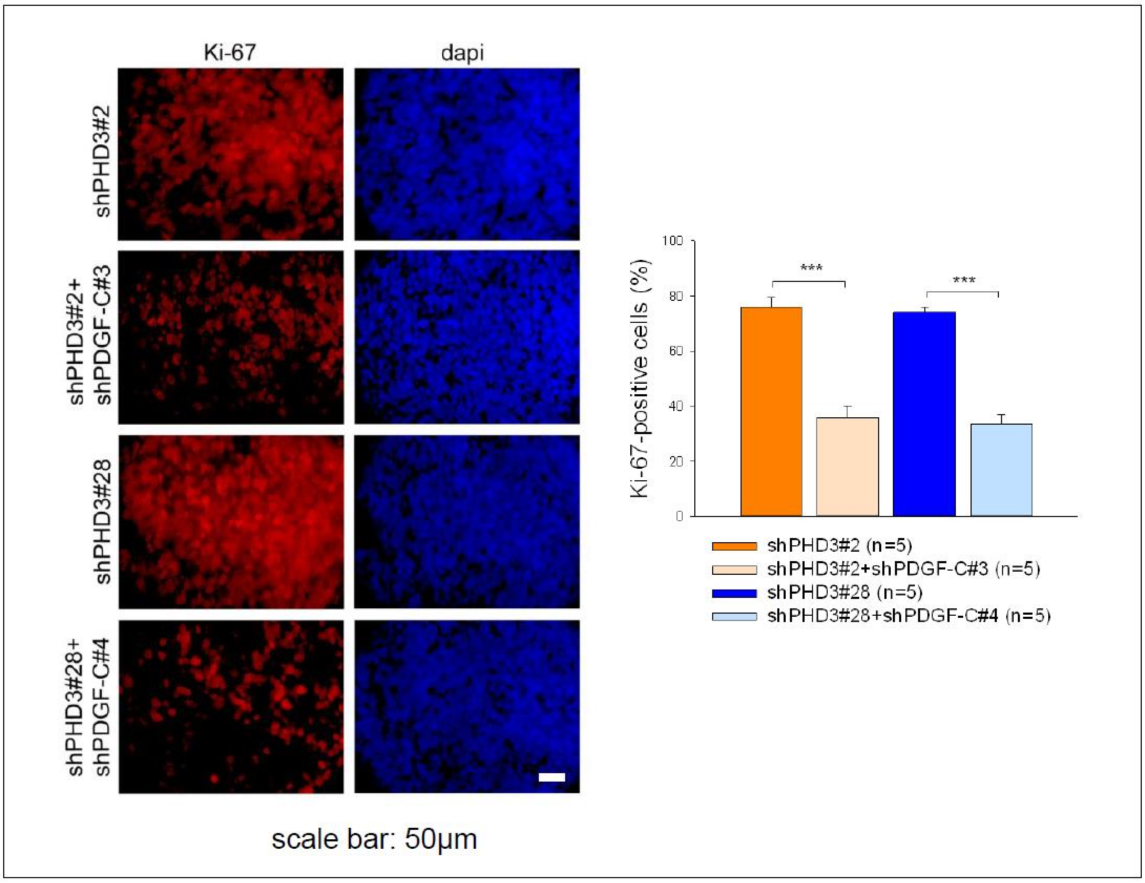

Figure S10. ShPHD3+shPDGF-C double know-down tumors are less proliferative compared to tumors in which PHD3 was silenced only.

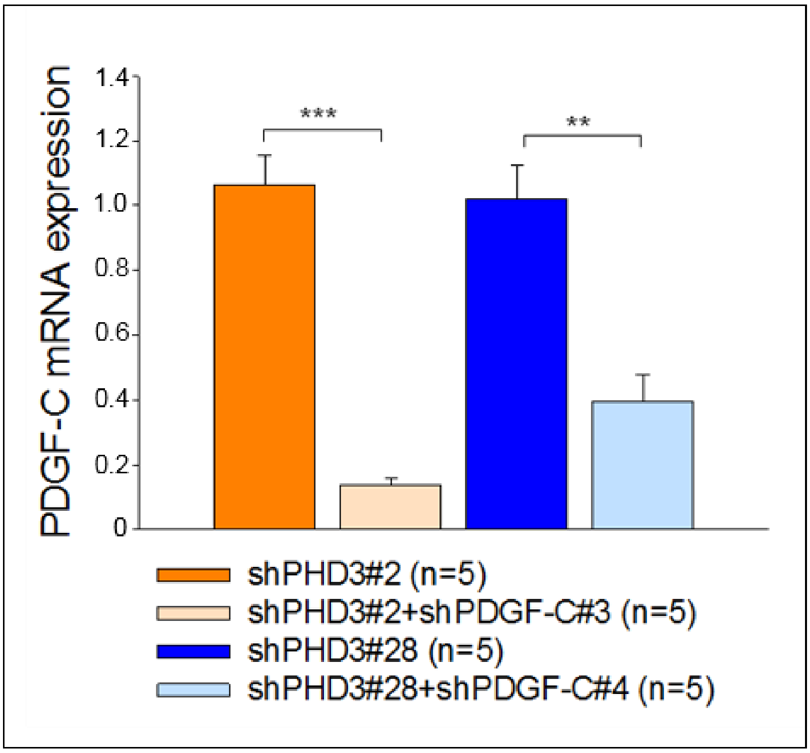

Figure S11. PDGF-C is significantly reduced in shPHD3 + shPDGF-C LM8 tumors.

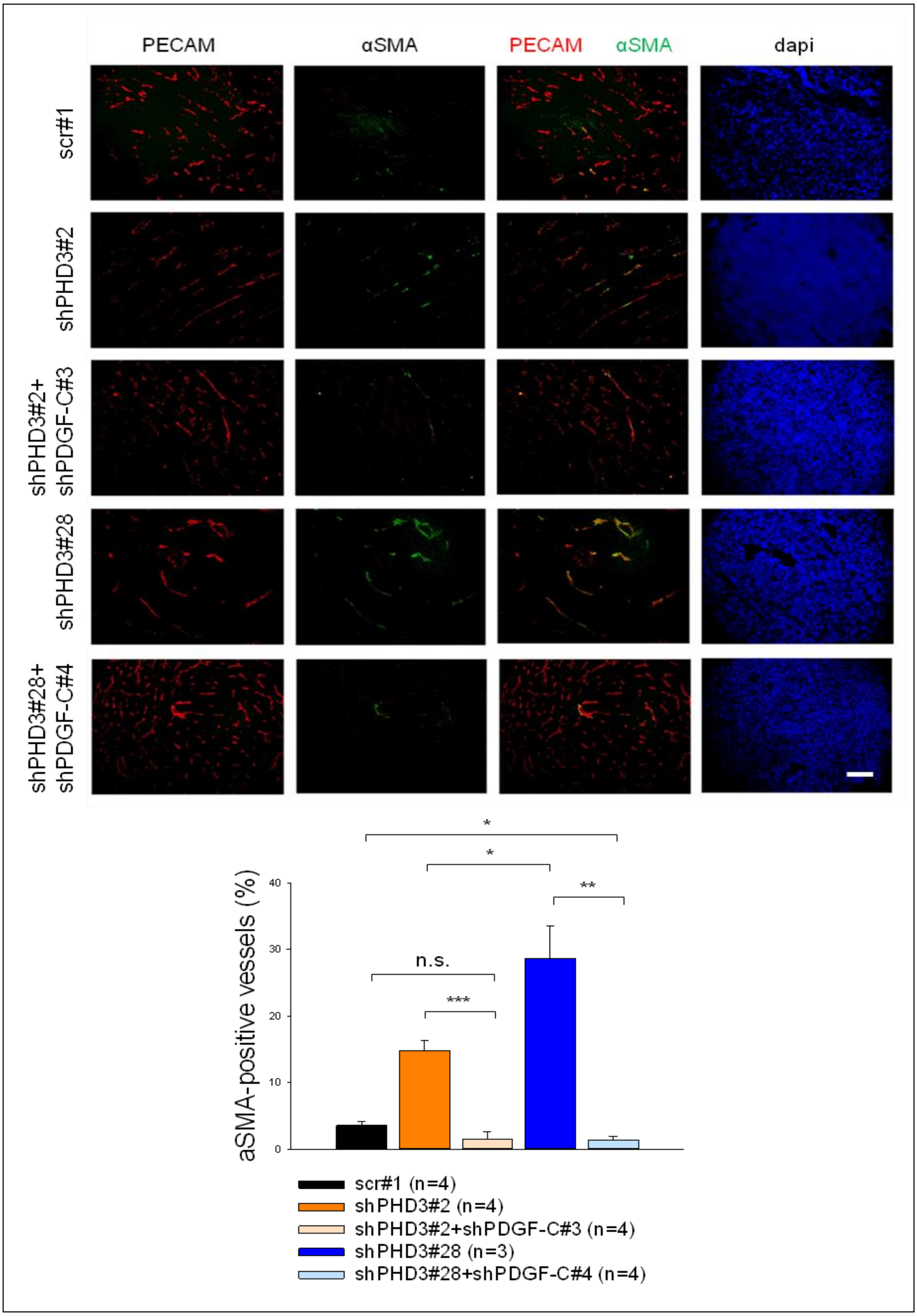

Figure S12. Knock down of PDGF-C in PHD3-deficient tumors reduces perivascular cell coverage of tumor vessels.

© 2018 by the authors. Licensee MDPI, Basel, Switzerland. This article is an open access article distributed under the terms and conditions of the Creative Commons Attribution (CC BY) license (http://creativecommons.org/licenses/by/4.0/).
